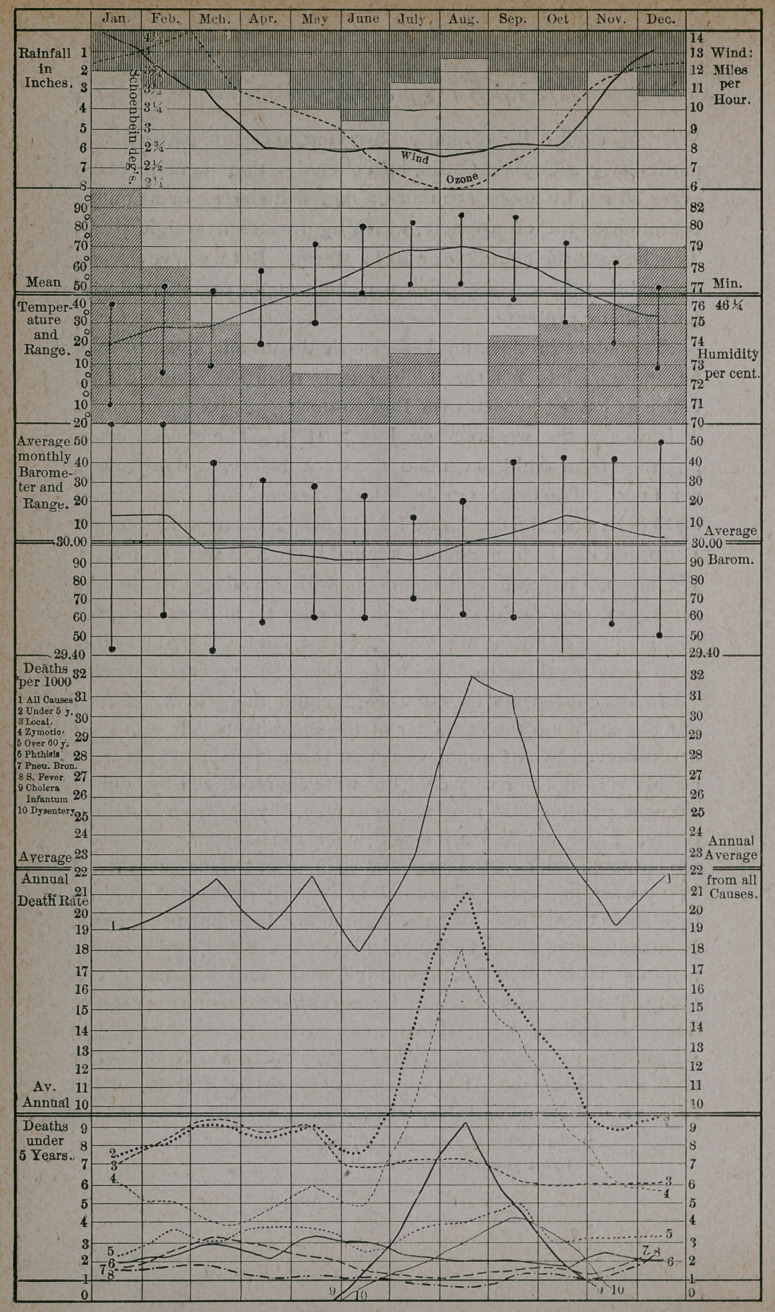# The Relation of Meteorology to Disease*Read before the Buffalo Medical and Surgical Association, Nov. 10, 1885.

**Published:** 1885-12

**Authors:** F. R. Campbell

**Affiliations:** Lecturer on Hygiene, Niagara University


THE BUFFALO MEDICAL AND SURGICAL JOURNAL

VOL. XXVI. DECEMBER, 1885. NO. 5.

### Original Communications.

### THE RELATION OF METEOROLOGY TO DISEASE.*

BY F. R. CAMPBELL, A.M., M.D.

Lecturer on Hygiene, Niagara University.

If it were the custom in our profession, as it is with the clergy, to choose from some ancient author a text for our discourses, I would select these words of Hippocrates: "Whoever wishes to investigate medicine properly, should proceed thus: In the first place, consider the seasons of the year and what effect each of them produces; then, the winds, the hot and the cold, especially such as are common to all countries, and such as are peculiar to each locality. And if it be thought that these things belong rather to meteorology, it will be remembered on reflection that astronomy contributes not a little, but a very great deal, to medicine; for with the seasons the digestive organs of men undergo a change." In another part of his work, Hippocrates gives an excellent account of the various meteorological conditions which preceded and accompanied certain epidemics. His followers paid so much attention to this subject, that Aristophanes in one of his comedies dubbed the

*Read before the Buffalo Medical and Surgical Association, Nov. 10, 1885.

medical profession of his time, "a pack of meteorological quacks." After the revival of learning, when physicians followed the letter rather than the spirit of the ancient authors, the rising of each star, and the signs of the zodiac, were supposed to have their effect upon the human body. Chaucer, in "The Nonnes Tale," says:

> "Ware the sonne in his ascencioun
> Ne fynde yow not replet of humours hote,
> And if it do, I dar wel laye a grote
> That ye shal have a fevere terciane."

Arbuthnot, in 1733, wrote a work upon this subject, entitled, "The Effects of Air on the Human Body," in which he pointed out the influence of seasons on disease, maintaining that each season is characterized by its special diseases. Dr. Prout, in his Bridgewater Treatise, recorded the first observations showing the relation of atmospheric pressure to disease, and claimed that there was a positive increase in the weight of the air when cholera first made its appearance in England.

Although so much studied in the past, etiologists have at present almost given up the investigation of atmospheric causes of disease. All are busy searching for specific germs; by their constant microscopical labors they have become intellectually myopic, and cannot see that there may be causes of disease which it is impossible to place beneath a cover glass. They insinuate that the study of medical meteorology is a subject redolent with the ignorance of the Dark Ages, and that he who is devoted to these investigations should be placed in the same category with the Irishman who wants his vernal purge and the German who annually submits to phlebotomy. A belief in germ theories of disease is not, however, in any way shaken by the study of atmospheric causes. The alkaloids found in a given species of plant vary in quality and quantity with the soil and climate in which they are grown. It will not be unreasonable, therefore, to suppose that bacteria, vegetable organisms so minute that a few days of culture in an altered medium will greatly modify their pathogenetic powers, will also be profoundly altered by the influence of various meteorological phenomena. A microbe, which under the influence of cold or an excess of ozone in the air may be perfectly harmless, will, by the effect of heat or other atmospheric change, generate a deadly poison, and perhaps cause an epidemic. The opinion of a botanist who would describe only the anatomy of a plant without telling us what soil and climate are most favorable to its growth, would not be highly valued; so the pathologist who informs us that the microbe of a given disease is shaped like a period, a dash, or a comma, without telling us what meteorological conditions are most favorable to its development, does only half his duty.

If we reject germ theories, the relation between atmospheric changes and disorders of human body is even more easily comprehended. The organic chemical processes in plants which depend entirely upon soil and climate for their development, have their analogies in the human system which are also influenced by meteorological changes.

The difficulties which surround the investigation of a subject such as this, are quite manifest. In the first place, we must study a number of constantly-varying factors, precipitation, temperature, pressure, ozone, etc., and, on the other hand, we must investigate a number of variable diseases not always depending upon the associated atmospheric conditions. For example, we observe that cases of diphtheria, scarlet fever and measles decrease in a very marked manner in June and increase again in October. From a meteorological point of view, we would say that this reduction is due to the high temperature. But the student of school hygiene will tell you that this results from the closing of the schools, the contagion being less easily propagated, while the "fresh air" enthusiast is convinced that the decline in the number of these cases is due solely to the fact that the windows are kept open at this season of the year. In the second place, the influence of metereological changes is usually indirect. We have a prolonged drought in a city where well water is largely used, and, as a result, typhoid fever and dysentery increase. A heavy rain follows the drought, soaking into a soil which was, perhaps, at one time a swamp. The malarial poisons which have for some time been accumulating, are thus displaced, and intermittent fevers prevail.

In cities, filth is common, especially with the poor. The decomposition of this dirt does not appear to be particularly injurious to adults who are long accustomed to it. Like Byron's Lisbonites:

> "The dingy denizens are reared in dirt,
> No personage of high or low degree
> Doth care for cleanness of surtout or shirt,
> Though shent with Egypt's plague, unkempt, unwashed, unhurt.

Yet, when the summer sun warms up this mass of corruption, the children and new-comers in those districts are swept away in such numbers by diarrhœal diseases, that the annual death rate in our city for some weeks has been as high as 49 per 1,000, a rate scarcely surpassed in the filthy cities of Mexico and Spain. Atmospheric changes alone, without the co-operation of other causes, would be comparatively inert. The death rate in Buffalo has, during the past four years, decreased at least fifteen per cent., and the reduction of the infant mortality has been even greater. We do not attribute this entirely to a gradual improvement in the weather, nor to the increased skill of our physicians; this has been brought about largely by the laying of water mains, the extension of sewers, and the general improvement in the sanitary condition of the city, which the Board of Health has endeavored to enforce.

It is on account of these difficulties that a scientific study of meteorological medicine has hitherto been impossible. When weather bureaus and vital statistics were unknown, no accurate conclusions could be reached. At the present day, however, all this is changed, a scientific study of medical meteorology is possible, and no country in the world has better facilities for this than the United States. The Signal Service furnishes daily, almost hourly, records of the weather, with weekly, monthly and annual summaries. Vital, or at least mortuary, statistics are accurately recorded in the majority of our large cities. From these data, we may expect in the near future the development of a science of medical meteorology.

In order to facilitate my investigation of this subject, I have prepared a series of charts deduced from the weekly mortuary statistics issued by the Health Department of Buffalo, and the weekly meteorological summaries furnished by the signal station located here. My observations have extended over a period of four years, from 1881 to 1884 inclusive. Vital statistics for the year 1880 are obtainable, but they are so inaccurate and incomplete as to be worthless for making comparisons. By a single glance at one of these charts, you can ascertain, for each week: (1) the amount of rainfall, (2) the velocity of the wind, (3) the range of the thermometer, (4) the mean temperature, (5) the range of the barometer, (6) the mean atmospheric pressure, (7) the rate of deaths from all causes, (8) the rate of infant mortality, (9) the death rate from zymotic diseases, (10) the death rate from local diseases, (11) the death rate from phthisis, (12) the death rate from pneumonia and bronchitis, (13) the death rate from scarlet fever, (14) the death rate from cholera infantum, (15) the death rate from dysentery and diarrhœa, and (16) the death rate for those over sixty years of age. By means of red lines running across the chart, you may learn the mean annual barometer and thermometer, and the average annual death rate from all causes and for those under five years of age.

I have also prepared a fifth chart, showing the average, for the four years, of the meteorological conditions recorded and the average of the death rates. To this chart I have added the average humidity and the average amount of ozone for each month, deduced from the average of a number of stations located on the same isotherm as Buffalo.

We will first discuss some of the general characteristics of our climate. The mean annual temperature has, during the past four years, been 46¼ degrees. Our climate is, therefore, according to Humboldt's classification, cold; since only those climates having a mean temperature of at least 50 degrees are considered temperate, the climate of New York City or Philadelphia for example. The highest temperature recorded during this period is 91 degrees, the lowest 14 degrees below zero, making an extreme range of 105 degrees, though the annual range is about 100 degrees. The diurnal range of the thermometer is sometimes as high as 35 degrees, though the average range is less than for places located further from the lakes. The changes in temperature here may be sudden, but not extreme, as has been observed in Texas, where the temperature has been known to fall from 83 to 22 degrees within 41 hours. The seasons are somewhat retarded, the mean weekly temperature rising above the annual average about the 1st of May and falling below it about the last of October. Our prevailing winds are west and southwest; their velocity is above the average, and, as they come from the lakes, the relative humidity is comparatively high, the average for the past four years being 75. It has been demonstrated that places near a lake, especially if the velocity of the wind is high, have an excess of atmospheric ozone. For this reason, which is supported by observations made in Michigan, we may safely assume that the air of Buffalo contains more than the average amount of this substance.

There seems to be a direct relation between the mean annual temperature, atmospheric pressure and humidity, and the mortality rates, as may be seen from the following table:

| YEAR. | Mean Annual Temperature. | Mean Annual Barometer. | Average Relative Humidity. | Annual Death Rate for all Diseases. | Annual Infant Mortality. | Annual Death Rate for those over 60 years of age. |
|---|---|---|---|---|---|---|
| 1881 | 47.3° | 30.024 | 73.9 | 25.2 | 11.9 | 3.52 |
| 1882 | 47. | 30.05 | 74.9 | 23.6 | 11.2 | 3.12 |
| 1883 | 44.9 | 29 98 | 74.4 | 19. | 8. | 3.07 |
| 1884 | 45.8 | 30.022 | 79.3 | 20.6 | 9. | 2.91 |
| Av'ge, | 46¼° | 30.019 | 75. | 22.2 | 10.0 | 3.16 |

From this table we may deduce the following propositions:

1. That the death rate in Buffalo increases directly with the mean annual temperature.

2. That for each degree that the mean annual temperature rises above 45 degrees, the infant mortality increases 1 per 1,000.

3. That the death rate for persons over sixty years of age diminishes as the mean annual relative humidity increases.

Observations extending over a period of but four years are not sufficient to demonstrate these propositions conclusively, but it is at least indicated that more danger to the public health is to be apprehended in a warm than in a cold year, and that this danger is especially grave for those under five years of age.

It is more difficult to explain the relation existing between the relative humidity and death rate for those over sixty years of age. I will offer, as an explanation, the fact that the diurnal range of the thermometer diminishes as the relative humidity increases; and it will be shown hereafter that sudden reductions of temperature are especially injurious to old people. The increased humidity of the air, by reducing the range of the temperature and making changes less sudden, renders the climate more suitable to the aged.

In discussing the influence of the seasons on disease, many atmospherical phenomena, besides change of temperature, should be considered. As the thermometer in our climate rises, the corrected barometer falls; the relative humidity of the air decreases, while the actual humidity becomes greater. In the hot season, the range of barometer and thermometer, the velocity of the wind and the relative humidity are at a minimum. With the slowing of the air currents and the diminished range of the temperature, the amount of ozone in the air is reduced, partly because less is generated, and partly because much is taken up by decaying vegetable matter, which is especially abundant at this time.

Let us first consider the effect of the seasons upon healthy persons. This subject has been carefully studied by Mr. Milnor, surgeon to a convict establishment at Wakefield, England. The prisoners on whom the observations were made were all males between the ages of fifteen and sixty and were usually in good health. Their cells were all of the same capacity; they all breathed the same air, had the same hours of labor and rest, and ate the same food. These men were weighed on admission and at the end of each calendar month, the observations being extended over a period of ten years. The total number of individual weighings was 44,004. It was found that there was an average loss during the first three months of the year: in January, 0.14; February, 0.24; March, 0.95 per cent. During the next five months there was an average gain as follows: April, 0.03; May, 0.01; June, 0.52; July, 0.08; August, 0.70 per cent. Then followed another period of loss: 0.21 in September, 0.10 in October, and 0.03 per cent. in December. There was a very slight gain in November, but this was attributed to the fact that there was a large increase in the admission at this time, and the convicts generally increased in weight shortly after entering the institution. From these observations, Mr. Milnor deduced the following inferences:

"1. That the human body becomes heavier during the summer months and the gain varies in increasing ratio.

2. The body becomes lighter during the winter months, and the loss varies in increasing ratio.

3. The changes from gain to loss are abrupt, and take place about the end of March and the beginning of September."

The influence of seasons on diseases, which varies greatly in different climates, is more easily studied and explained. Haviland observed that diseases resembled plants in this particular; some diseases were annuals, some deciduous, and others perennials. In our climate, we have no well-marked disease of the "annual" type. Yellow fever, which springs up with the summer heat to disappear with the first hard frost of autumn, is a good example. Small-pox, with us, has behaved like an annual, but this is due to the fact that our health authorities treat it much as the gardener does his weeds, and we need only glance at its history, before vaccination became general, to recognize it as a very hardy perennial.

The diarrhœal diseases of children, generally classified as "cholera infantum" in our mortuary statistics, illustrate, in a remarkable manner, the "deciduous" class of diseases. As regularly as the roses bloom in June, cholera infantum follows in their path, furnishing a theme for the poets and work for the doctors. Infantile diarrhœa makes its appearance in June, increases through July, culminating during the first part of August, and dies away in October to await the coming of another summer with its roses and fresh supply of babies. The majority of diseases in Buffalo are decidedly "perennial" in their type. But just as the pine puts on its greenest foliage at a certain period of the year, diseases seem to have their periods of greatest severity. In order to investigate this subject, it is convenient to divide the year into quarters of thirteen weeks each. I give below a table showing the average death rate per 1,000 for several diseases and classes of diseases as derived from the vital statistics of Buffalo for the years 1881 to 1884 inclusive:

| Quarter. | First. | Second. | Third. | Fourth. | Average. | Percentage of all Deaths. |
|---|---|---|---|---|---|---|
| Death rate per 1,000 from all causes | 20.3 | 19.7 | 28. | 20.75 | 22.2 | .............. |
| Death rate per 1,000 under 5 years of age | 8.3 | 7.41 | 17.60 | 9.30 | 10. | 45 per cent. |
| Deaths per 1,000 from zymotic diseases | 4.8 | 4.95 | 13.10 | 6.90 | 7.44 | 33⅓ per cent. |
| Deaths per 1,000 from constitutional diseases | 3.5 | 3.33 | 2.9 | 4.11 | 3.26 | 14.7 per cent. |
| Deaths per 1,000 for local diseases | 8.4 | 6 .9 | 7.27 | 6.61 | 7.30 | 33 per cent. |
| Deaths per 1,000 from scarlet fever | 1.55 | 1.25 | .91 | 1.52 | 1.21 | 5½ per cent. |
| Deaths per 1,000 from diphtheria | 1.10 | .30 | .40 | 1.44 | .81 | 3 6 per cent. |
| Deaths per 1,000 from pneumonia and bronchitis | 3.05 | 2.71 | 1.30 | 1.90 | 2.25 | 10 per cent. |
| Deaths per 1,000 from phthisis-pulmonalis | 2.02 | 2.51 | 2.20 | 2.02 | 2.19 | 9.9 per cent. |
| Deaths per 1,000 from cholera infantum | .... | .13 | 6.55 | .56 | 1.81 | 8.2 per cent. |
| Deaths per 1,000 from dysentery and diarrhœa | .... | .23 | 2.63 | .82 | .92 | 4.2 per cent. |
| Deaths per 1,000 for those over 60 years of age | 3.06 | 3.10 | 3.19 | 3 23 | 3.16 | 14 2 per cent. |

It may not be uninteresting to note the differences of the relation of seasons to disease depending upon climate and location in cities widely separated.

| City. | Per Cent. of Deaths. | | | |
|---|---|---|---|---|
| | 1st Quarter. | 2d Quarter. | 3d Quarter. | 4th Quarter. |
| Buffalo................ | 23 1 | 22.2 | 31.5 | 23.2 |
| London............ . | 25. | 21. | 24. | 28. |
| St. Louis.... ........... | 22.7 | 22.3 | 29. | 26. |

In all these places, the second quarter appears to be the healthiest period of the year; but on the other side of the Atlantic, there is a gradual increase in the death rate from June to December, while with us the healthiest period is immediately followed by the most sickly. Diarrhœal diseases belong more distinctly to the summer months in Buffalo than is the case in warmer climates, and, indeed, there are but few cities with so low a mean temperature as ours in which diarrhœal diseases assume so nearly an epidemic form in summer. It is with some hesitation that I attribute this high mortality to the excessive filth and defective sewerage of some portions of our city; yet, when we observe that the mortality from diarrhœal diseases is greatest in those parts of the city where there is the most filth and the poorest system of sewerage, we can ascribe it to no other causes.

In the first quarter of the year, the weekly range of the thermometer and barometer, the mean atmospheric pressure, the relative humidity and the amount of atmospheric ozone, are at a maximum, the velocity of the wind is above the average and the mean temperature is at its minimum.

In this quarter, the class of local diseases attains its maximum, pneumonia and bronchitis being most prevalent in March, when the winds are high and the temperature is oscillating above and below the freezing point. Scarlet fever is also at its maximum in this quarter, while diphtheria and constitutional diseases are above the average. Zymotic diseases, as a class, are now at their minimum, while the mortality from phthisis is below the average. Fatal cases of diarrhœal diseases are in this quarter almost unknown.

In the second quarter, the rainfall reaches its maximum; the mean atmospheric pressure, the minimum. The temperature ranges from 55 to 65 degrees. The amount of ozone in the air is about the average for the year. The average velocity of the wind is quite uniformly nine miles an hour; the relative humidity is about seventy-five per cent., or just equal to the annual average. Only one disease attains its maximum death rate in this quarter, viz.: phthisis; but pneumonia, bronchitis and scarlet fever are above the average. The average annual death rate reaches its minimum from the twentieth to the twenty-sixth week, that is, from the middle of May to the last of June. At the same time, the infant mortality and the death rate for diphtheria are at their minimum, and diarrhœal diseases begin to appear.

In the third quarter, the temperature reaches its maximum, the range of thermometer and barometer, the rainfall, the velocity of the wind, the relative humidity and the amount of atmospheric ozone reach the minimum. The average annual death rate reaches its maximum about the thirty-fourth week, or about the last of August. If, however, there were no deaths from diarrhœal diseases, this would be the healthiest quarter of the year. The maximum infant mortality, and the greatest death rate for cholera infantum, occur in the first week of August, when the temperature is at its maximum. The maximum death rate for dysentery is reached a month later, in September, at a time when the effects of drought are most felt, and the diurnal range of barometer and thermometer have increased. The mortality from scarlet fever, pneumonia, bronchitis and the constitutional diseases, as a class, reaches the minimum, and the deaths from diphtheria are much below the average.

In the fourth quarter, the range of barometer and thermometer, the relative humidity, the velocity of the wind and the mean cloudiness, are above the average; the temperature is below the annual average, and subject to frequent variations. In this quarter occur the greatest number of deaths from constitutional diseases as a class, from diphtheria, and of persons over sixty years of age. The deaths from scarlet fever are greatly above the average; diarrhœal diseases are disappearing, and the mortality from phthisis is below the average. It is interesting to nôte that the death rate for consumption in our city is greatest in the spring and summer months, and not in the fall and winter, as is generally supposed.

We will now discuss the effects of some of the common meteorological phenomena in detail:

1. *Precipitation or Rainfall.* In our climate there is always an excess of cloudiness with an excess of rain, though this is not the case in all places—the elevated regions of Colorado, for example. We are obliged, therefore, to consider the effects of deficient sunlight in connection with rainfall. Excessive rainfall in our country does not appear to be particularly injurious to the public health. In poorly-drained districts, and where well water is extensively used, excessive rains have been known to cause malarial fevers and diarrhœa; but a deficient rainfall is especially dangerous, as has often been illustrated by the history of epidemics. The plagues which have visited China have generally been preceded by drought, as was also the case with the pestilence at Constantinople in 1541. The greatest death rate in England, during twelve years, appeared, according to Steinmetz, in the year with the least rainfall, in 1864. In Buffalo, in 1881, there was a protracted drought. During six weeks in August and September, less than a quarter of an inch of rain fell; the atmosphere became hazy, the wells were dried, in some the odor of sewage was very perceptible, and the soil was parched to a depth of five feet. The maximum death rate, for the past five years at least, was reached at this time. For two weeks in September, it remained at 49 per 1000. Dysentery became epidemic, twenty-five per cent. of all deaths being due to this cause, while most of the other zymotic diseases assumed a more virulent type. About the first of October, heavy rains set in and dysentery immediately began to disappear. In no other year studied have we had so severe a drought, and in no other year has dysentery been more than half so fatal.

From observations made upon the effect of heavy rains upon the death rate, the following conclusions have been reached, though there are some exceptions:

(1) That a fall of rain, when the mean temperature is above 55 degrees, will reduce the death rate.

(2) That a fall of rain, at other times, will increase the death rate.

(3) That a fall of snow has no effect upon the death rate.

2. *Humidity.* I am informed, by Sergeant Cuthbertson, that the relative humidity of the air in Buffalo is higher than that of most cities along the Great Lakes, except the cities of Michigan. When we have westerly or southerly winds, the relative humidity increases. The relative humidity reaches its maximum in January, declines gradually to its minimum in August, and then rises again until January. During very cold weather, the absolute humidity is very low; as a consequence, when active exercise is taken at such times, the respiratory mucous membranes become dry and parched — an experience with which we are all familiar. When a warm room is entered after such exercise, re-action sets in, predisposing to congestion and catarrh. If the temperature is higher and the winds blow from the lake, the radiation of heat from the body is increased, since moist air will cool the body more rapidly than dry. This also predisposes to local congestions, and it is for these reasons that pneumonia, bronchitis and influenza are most fatal at this time. Cold with dryness, which is characteristic of the first period of the year, is not so injurious to consumptives as the cold, moist air, which comes a little later in the year. In New York City, with a mean temperature seven degrees higher than ours and a higher relative humidity, fourteen per cent. of all deaths are due to phthisis, while in Buffalo only ten per cent. are due to this cause. In other words, there is twenty-nine per cent. less phthisis in Buffalo than in New York.

Lastly, moist air is a better carrier of zymctic poisons than dry air. The dry Harmattan wind which blows from the interior of Africa, it is asserted, puts an end to all zymotic diseases, even small-pox, and interferes with the operation of vaccination. On the other hand, the majority of zymotic diseases are most prevalent when there is a high humidity. Small-pox, measles, scarlet fever and diphtheria all reach their maximum when the relative humidity is above the average. During the epidemic of dysentery in 1881, although there was a drought, the relative humidity was 69.3 ; and when we consider the high temperature which prevailed at this time, the absolute humidity must have been above the average for that month. The amount of carbon dioxide eliminated by respiration is greater in moist air than in dry, as has been demonstrated by the experiments of Paul Bert. This may afford some clue to the explanation of the fact that there are fewer deaths from old age in moist years than in dry, since tissue change and animal heat are more easily sustained when watery vapor is present in the respired air.

3. *Atmospheric Pressure.* The range of the barometer in Buffalo during the past four years has been about 1.60 inches. During the hot season, the actual atmospheric pressure is below the average. The range of the barometer is greatest in the colder months and diminishes as the weather becomes warm.

The effects of increased atmospheric pressure have been studied among those who work in caissons and those treated for disease by compressed air. No unpleasant effects, except possibly ringing in the ears, are felt while the pressure is increased. A high atmospheric pressure increases mental and physical vigor. Dr. B. W. Richardson explains this by assuming that the circulation of blood in closed cavities is increased by the high external pressure. Whether this be true or not, the rapidity of the pulse and respirations are reduced by high pressure, so that it requires less effort to sustain life under such conditions.

When the pressure is again reduced, unpleasant symptoms come on. With those who have the caisson disease described by Dr. A. Smith, severe neuralgic pains in the extremities, associated sometimes with pain in the epigastric region, and spinal congestion are observed. Pains of a similar character are often noticed during the period of low pressure which precedes a storm. A low pressure in our climate is usually associated with increased humidity, and the exhalation of watery vapor becomes more difficult, perspiration easily collects on the surface of the body on account of the slow evaporation, and a general feeling of lassitude comes over us. The respirations and pulse become increased, partly because expansion of the lungs is more difficult and partly because there is less oxygen in a cubic foot of air than when the pressure is higher. Pulmonary hemorrhage is sometimes produced by a rapid reduction of the atmospheric pressure — a fact which has been proved by those who have ascended mountains. Invalids sent to the elevated regions of Colorado are often obliged to make a very gradual ascent, or their disease will become aggravated. It is quite probable that most consumptives would do better if they would find a dry atmosphere without a reduced pressure. In 1882, we had the highest annual barometer, 30.05, and the lowest death rate from phthisis, 2.01 per 1,000, or 8½ per cent. of the total number of deaths. In 1883, we had the lowest annual barometer, 29.98, characterized by a high and frequently-changing range. The death rate for phthisis this year, although the general death rate was the lowest for four years, was the highest attained, 2.64 per 1000, or 13.9 per cent. of all deaths. In other words, the death rate from phthisis increased 31 per cent. in a single year. This change was not due to the moisture of the air, for the relative humidity was about the same for the two years, being 74.9 in 1882 and 74.4 in 1883. We do not forget that the temperature in 1883 was unusually low, but there is no good evidence that cold will increase the deaths from phthisis, since there is less of this disease here in winter than in summer, and less in cold climates than in the temperate. Rapid reductions of pressure are most injurious to the aged. Whenever there is a sudden fall in the barometer, the death rate for those over sixty years of age quite regularly increases, either in the week in which the fall takes place or in the week following. The explanation has been offered, that a lowering of the atmospheric pressure increases the pressure of the blood on the walls of the vessels and upon the tissues, thus predisposing to apoplexy, paralysis and inflammation.

Surgical fever and the mortality from surgical operations are, at the present day, supposed to depend principally upon the skill of the operator and the dressings employed, yet it may not be uninteresting to note that Dr. A. Hewson, of Philadelphia, a few years ago, published a paper in which he claimed that atmospheric pressure had a marked influence upon the results obtained. His observations extended over a period of thirty years in the Pennsylvania Hospital, and he found that the mortality from capital operations was 10.7 per cent. with a rising barometer, 20.69 with a stationary barometer and 28.4 per cent. with a falling barometer.

4. *Temperature.* The effects of continued high temperature are strikingly illustrated in our climate by infantile diarrhœal diseases. Cholera infantum appears when the mean temperature rises above sixty degrees, or about the 1st of June. During the next four or five weeks, the death rate is not high, but in July the disease rapidly increases in severity, reaching its maximum about the first week in August, at which time the excess of accumulated heat is greatest. During the hot season, the diurnal variation in the relative humidity is nearly four times as great as during the cold season. During the heat of the day, much watery vapor is taken into the air; at night the temperature falls, and the relative humidity approaches saturation. In the middle of the afternoon, the relative humidity is lowest, perspiration is abundant, but perhaps insensible, and the sweat glands are highly stimulated. At night, when the humidity of the air is very high, perspiration is difficult, and the elimination of fluid is turned into a new channel—the intestinal tube. It is for this reason, probably, that diarrhœal diseases and cholera morbus are so liable to begin at night. Infants are more easily affected by heat than adults, cholera infantum appearing from ten to twenty days earlier in the year than dysentery and cholera nostras. During the first two months of infantile life, the sweat glands have but little functional activity, and it is not until the child is five years old that the sudoriparous functions are fully established. Hence, when we have great heat, the temperature of the child's body cannot be equalized, as it is by evaporation of perspiration in the body of the adult, and fluids must be eliminated by the lungs, kidneys and bowels. We do not overlook the fact that choleraic diarrhœa is most common in filthy and badly-drained districts, but the action of heat is necessary before the diarrhœa appears. In *L'Abeille Medicale* for Oct. 26th, there is published a communication to the French Academy of Medicine by Dr. Le Bon, in which he maintains that cholera Asiatica and cholera nostras are due to precisely the same cause—volatile ptomaines—which he has demonstrated in the air over a "Sacred Pond" in India. The great heat, acting upon the masses of decomposing filth about this pond, generrates the poison, which, if inhaled for a certain period, will produce cholera Asiatica, if for a shorter period, the disease produced will be cholera morbus; that is, the disease produced depends upon the dose of the volatile ptomaine. M. Le Bon considers bacteria the carriers of this poison.

A high temperature diminishes the activity of certain zymotic poisons, notably those of scarlet fever, diphtheria, cerebro-spinal meningitis and small-pox. It was observed in Calcutta, that twenty-one per cent. of all vaccinations failed during the hot season, while but two and a-half per cent. failed during the cold. The plague in Egypt generally disappears when the mean temperature reaches eighty-two degrees. Phthisis diminishes with great heat as well as with great cold. In Buffalo, the lowest mortality occurs in the coldest months; in St. Louis, the lowest mortality is found in the hottest months, as may be seen from the following table:

| City. | Per Cent. of Deaths from Phthisis. | | | | |
|---|---|---|---|---|---|
| | First Quarter. | Second Quarter. | Third Quarter. | Fourth Quarter | Deaths per 1,000. |
| Buffalo.......... | 23. | 28.7 | 25.1 | 23 2 | 2.19 |
| St. Louis........ | 26.4 | 27.7 | 21.1 | 24.4 | 2.13 |

It is, perhaps, a mistake, therefore, to send our consumptive patient south to avoid "our rigorous northern winters," when the winter death rate for phthisis is even greater in the south than with us. It would probably be just as well to send these patients southward in the summer and northward in the w nter, thus keeping them beyond the danger lines; though, if this were done, they might die of diarrhœa or pneumonia.

Sudden changes in temperature have quite a different effect upon diseases than a gradual rise or fall. Tardieu states that for each degree that the temperature of the air is increased, the temperature of the body rises one-twentieth of a degree. For example, if the thermometer should suddenly rise from 60 to 80 degrees, the temperature of the body would rise one degree. This statement is confirmed by the experiments of Becher, Eydaux and Brown-Sequard. If the air is moist, the amount of carbon dioxide eliminated will at the same time be increased. A sudden rise of temperature, then, tends to produce inflammatory diseases, especially in children. March, 1882, was characterized by sudden changes in temperature, and the mean temperature was 2½ degrees warmer than the average for that month. We find at this time a great increase in the mortality from pneumonia and bronchitis, the death rate reaching 7 per 1000—about three times greater than the average. I have tabulated forty-eight sudden changes of temperature, which demonstrate this point:

| Change. | Increase of Infant Mortality. | Increase of Mortality of Aged | No Effect. |
|---|---|---|---|
| Rise, 19 observation........... | 18 times. | 10 times. | 1 |
| Fall, 29 observation........... | 10 " | 19 " | 8 |

From my observations in this particular, I conclude:

(1) That a sudden rise of temperature, especially when the mean temperature is below the mean annual temperature, is more dangerous to the public health than a sudden fall.

(2) That a sudden rise of temperature is most dangerous to the health of infants.

(3) That a sudden fall of temperature is most dangerous to the health of the aged.

Dr. Wm. Farr, the English sanitarian, from a careful study of the vital statistics of England, has formulated the following general law: "*After a man has reached his twentieth year, the danger of dying from a sudden fall of temperature is doubled every nine years.*" It is quite probable, however, that the aged are injured more than the infants, because the former get out of doors, and thus an opportunity is afforded for them to become chilled. When they enter the house again, the sudden warmth produces an excessive flow of blood, and congestions, apoplexy, etc., are the result.

5. *Electrical Phenomena and Ozone.* Air that has been breathed becomes unable to sustain life, even though all the carbon dioxide and organic impurities are carefully removed. This has been experimentally demonstrated by Dr. B. W. Richardson, who found that even pure oxygen, after having been re-breathed a number of times, would not sustain life in rabbits, and lost its oxydizing power. If, however, an electric spark be passed through oxygen thus rendered inert, it regains its power of sustaining life. This process of electrifying oxygen is continually going on in nature, for the movements of the air, the changes of temperature, and the fluctuation of the atmospheric moisture, all generate electricity. This electrified oxygen is in part ozone, but the amount present in the air is constantly varying with the season, the winds, and time of day. The greatest amount of ozone in the air is found between 9 P. M. and 7 A. M., the least between 2 P. M. and 7 P. M. During the day, the electric currents usually pass from the earth to the clouds; shortly after sunset, the currents pass in the opposite direction; toward morning, the currents are nearly stationary. There is, undoubtedly, some relation between the movement of these electric currents and the nocturnal pains of syphilis, rheumatism and some other diseases.

In discussing the relation of atmospheric ozone to disease, we are obliged, as I have before stated, to assume that the amount of ozone in the air of Buffalo is the same as the average for Michigan.

The maximum amount of atmospheric ozone is found during the latter part of February. From this time there is a steady decline in the amount until August, when the minimum is reached, after which there is again a rise until February. When the amount of atmospheric ozone is greatest, we have the maximum of acute respiratory diseases and the minimum of intestinal diseases; when the amount of atmospheric ozone is least, we have the maximum amount of intestinal diseases and the minimum of acute respiratory diseases. There is a greater amount of ozone in the air of places near bodies of water, especially salt water. If ozone is a cause of pneumonia and bronchitis, then we have a right to expect the death rate from these diseases to be greater in seaport towns than in the interior. This appears to be true, as may be seen from the following table:

| City. | Deaths per 1,000 | Percentage of all Deaths |
|---|---|---|
| New York | 3¼ | 14 |
| San Francisco | 2⅓ | 11 |
| Buffalo | 2¼ | 10 |
| St. Louis | 1½ | 6 |

This may be, in part, due to the high winds of seaport towns, since high, moist winds rapidly abstract heat from the body.

The relation of the electrical phenomena, associated with storms, to nervous diseases has been carefully studied by Dr. S.

Weir Mitchell. By carefully observing neuralgic patients, and particularly a Captain Catlin, he concludes that there precedes every storm, by about 150 miles, a modified electric or magnetic condition of the atmosphere, which aggravates neuralgic pains. This he calls the neuralgic area of a storm. But we have already noticed that these same pains may be brought on by a reduced atmospheric pressure.

Tornadoes produce marked electric disturbances, which may be prejudicial to health. Only one is recorded at the Signal Office as having passed over Buffalo during the past four years. This was in the week ending July 7, 1883. The general death rate rose from eight per 1,000, the week before, to twenty-one per 1,000. The death rate from local diseases rose from two to eight per 1,000, the death rate for old people from .30 to 3.60 per 1,000, and deaths from pneumonia and bronchitis were increased. We have not had sufficient experience with tornadoes to make any accurate deductions, but as the death rates for all these diseases fell again the following week, it is, at least, indicated that this tornado had something to do with the increased number of deaths.

In conclusion, let us say a word about the general effect of our climate upon the character of the inhabitants. It was long ago observed by anthropologists that those climates which were neither very hot nor very cold, but were characterized by sudden changes, produced the most vigorous men, physically and intellectually. In ancient Sparta, it is said, where the balmy air constantly tended to effeminacy, the magistrates, in order to rear up a race of hardy warriors, destroyed the sick and poorly-developed children by placing them upon a deserted mountain. In a climate like ours, Nature assumes the task of the Spartan magistrates, and removes from our midst the weak, the sickly and the decrepit, thus developing a hardy race, unsurpassed by any either to the north or south. Those who attain manhood in our climate will endure the greatest vicissitudes in the burning Tropics or in the frozen North better than any other race. But we cannot recommend Buffalo as a health resort for invalids, nor inform our guests that—

> "The immortal gods purge all infection from our air,
> While you do climate here."

Jan. Feb. Mch. Apr. May June July Aug. Sep. Oct Nov. Dec.

Rainfall in Inches.

Schoenbein deg's.

Wind

Ozone

Wind: Miles per Hour.

Mean

Min.

46¼

Temper-ature and Range.

Humidity per cent.

Average monthly Barometer and Range.

30.00

29.40

Average 30.00 Barom.

Deaths per 1000

1 All Causes

2 Under 5 y.

3 Local,

4 Zymotic

5 Over 60 y.

6 Phthisis

7 Pneu. Bron.

8 S. Fever

9 Cholera Infantum

10 Dysentery

Average Annual Death Rate

## Figures and Tables

**Figure f1:**